# Microfluidic Synthesis, Control, and Sensing of Magnetic Nanoparticles: A Review

**DOI:** 10.3390/mi12070768

**Published:** 2021-06-29

**Authors:** Roozbeh Abedini-Nassab, Mahrad Pouryosef Miandoab, Merivan Şaşmaz

**Affiliations:** 1 Department of Biomedical Engineering, University of Neyshabur, Neyshabur 9319774446, Iran; 2 Department of Electrical Engineering, University of Neyshabur, Neyshabur 9319774446, Iran; mahradpuryusef@gmail.com; 3 Department of Electrical and Electronic Engineering, Faculty of Engineering, Adiyaman University, Adiyaman 02040, Turkey; msasmaz@adiyaman.edu.tr

**Keywords:** microelectromechanical systems, MEMS, microfluidics, lab-on-a-chip, magnetic nanoparticles, nanoparticle synthesis, droplet microfluidic, microchamber, magnetic sensors, magnetoresistive, magnetic manipulation, magnetic transport, GMR, TMR, MRX

## Abstract

Magnetic nanoparticles have attracted significant attention in various disciplines, including engineering and medicine. Microfluidic chips and lab-on-a-chip devices, with precise control over small volumes of fluids and tiny particles, are appropriate tools for the synthesis, manipulation, and evaluation of nanoparticles. Moreover, the controllability and automation offered by the microfluidic chips in combination with the unique capabilities of the magnetic nanoparticles and their ability to be remotely controlled and detected, have recently provided tremendous advances in biotechnology. In particular, microfluidic chips with magnetic nanoparticles serve as sensitive, high throughput, and portable devices for contactless detecting and manipulating DNAs, RNAs, living cells, and viruses. In this work, we review recent fundamental advances in the field with a focus on biomedical applications. First, we study novel microfluidic-based methods in synthesizing magnetic nanoparticles as well as microparticles encapsulating them. We review both continues-flow and droplet-based microreactors, including the ones based on the cross-flow, co-flow, and flow-focusing methods. Then, we investigate the microfluidic-based methods for manipulating tiny magnetic particles. These manipulation techniques include the ones based on external magnets, embedded micro-coils, and magnetic thin films. Finally, we review techniques invented for the detection and magnetic measurement of magnetic nanoparticles and magnetically labeled bioparticles. We include the advances in anisotropic magnetoresistive, giant magnetoresistive, tunneling magnetoresistive, and magnetorelaxometry sensors. Overall, this review covers a wide range of the field uniquely and provides essential information for designing “lab-on-a-chip” systems for synthesizing magnetic nanoparticles, labeling bioparticles with them, and sorting and detecting them on a single chip.

## 1. Introduction

Nanomaterials have recently attracted enormous interest in various disciplines. They are an interesting class of materials with amazing magnetic, catalytic, mechanical, electrical, and optical properties, which are not achievable in bulk materials [1]. Magnetic nanoparticles have a great impact in various fields, including data storage, chemistry, biology, and nanomedicine [2,3,4,5,6,7,8]. They have attracted great interest in the scientific community over the past decade due to their novel magnetic properties and promising applications. These nanoparticles represent superparamagnetism, exchange bias, surface irregularity, etc. 

Biologists have used magnetic nanoparticles in applications, including but not limited to (i) diagnostic purposes when conjugated with antibodies; (ii) magnetically labeling biological particles for sorting purposes; (iii) targeted drug delivery when loaded with drugs; (iv) magnetic hyperthermia; and (iv) magnetic resonance imaging (MRI). 

Microfluidic systems offer a new-fashioned technology for manipulating fluids and tiny particles. The high accuracy, automation, and control provided by the microfluidic chips result in better material handling, cost efficiency, portability, lower raw material consumption, and more reproducibility [9,10,11,12,13]. With enhanced microfabrication technologies, it is possible to integrate multiple parts into a single microelectromechanical system and enable complicated tasks, such as automated continuous and sequential flows, separation, mixing, and so on [11]. Thus, microfluidic chips not only serve as novel nanoparticle production tools but also, together with magnetic nanoparticles, offer great opportunities in biotechnology.

Some conventional nanoparticle synthesis methods are co-precipitation, sol-gel, ultrasonication, sonochemical processing, thermal deposition, gas-phase synthesis, plasma, microwave irradiation, spray pyrolysis, laser pyrolysis, mechanical milling, and arc discharge. The unique properties of the nanoparticles are highly related to their size and morphology. Thus, it is crucially important to control their production parameters; however, achieving this control in the conventional bulk processes is usually challenging, and they may suffer from extensive batch-to-batch variability. Microfluidic systems, including continuous-flow microreactors and droplet-based microreactors, are considered modern tools for synthesizing magnetic nanoparticles with major improvements. However, researchers are still dealing with fundamental goals, such as large-scale microfluidic-based nanoparticle production, high-throughput self-assembled magnetic nanoparticle synthesis, and controls over the composition; size; and morphologies of the magnetic nanoparticles.

Manipulating magnetic nanoparticles, microbeads composed of magnetic nanoparticles, and magnetically labeled bioparticles in lab-on-a-chip systems have many biomedical applications. For example, magnetic particles can separate or sort bioparticles of interest. However, one important application is in the field of single-cell analysis. It is now widely accepted that the traditional biological analyses at the bulk level cannot detect cell heterogeneity in the biological samples [14]. Thus, studying rare cells which play key roles and define patient destiny in cancer or infectious diseases is challenging. As a result, scientists are moving towards single-cell analysis, where genotypes and phenotypes at the single-cell level are studied [15,16,17]. Another set of crucial biomedical applications of magnetic nanoparticle manipulation is microfluidic-based diagnostics, where measuring biomarkers in blood, urine, or serum provides essential information about diseases [18,19]. Sometimes, the biomarker concentration is low in body fluids, and its detection using conventional techniques becomes challenging. Thus, either tissue samples from the primary organ using invasive methods (e.g., biopsy) or more advanced methods capable of detecting rare biomarkers are required. Magnetic particles have brought desirable answers to this problem by purifying and detecting nucleic acids and proteins at low concentrations. Although researchers have achieved interesting results in magnetic particle manipulation for single-cell analysis and biosensor applications, the field is in its infancy, and improvements are needed. Scientists deal with challenges, including the chip surface passivation, control at the single-particle resolution, chip microfabrication cost, and biodegradation of magnetic nanoparticles.

Immunoassays detect target analytes (e.g., proteins) based on the specific interaction between a target antigen and an antibody. One of the most widely used assays is the sandwich immunoassays, in which the capture and detection antibodies “sandwich” the target antigen. The capture antibodies are immobilized on a substrate and are exposed to the sample of interest. The target antigen in the sample bind to the specific antibodies, and then the structure is exposed to the detection antibodies equipped with a label, forming the sandwich complex. In the end, typically after a wash step, the presence of detection antibodies depicts the availability of the target antigen in the sample. Magnetic nano-/microparticles with a large surface-to-volume ratio and functionalized with capture antibodies can enhance the efficiency of the mentioned assays. Moreover, it is possible to conjugate magnetic particles with detection antibodies and transport them to an antigen-coated chip. Although magnetic properties in the mentioned assays play a role for detection purposes (e.g., in magnetoresistance sensors) [19,20], they can also do the manipulation and separation tasks in sensors [19,21]. Important examples of immunoassays in which magnetic nanoparticles bind to the bioparticle of interest and immobilize on a surface, to be detected by magnetic sensors, are broadly available [22,23,24]. In addition, similar sensing methods can characterize the magnetic nanoparticles and evaluate their magnetic properties [3]. Researchers in various labs are working to enhance the sensitivity of the magnetometers and lower their noise to achieve the goal of detecting nanoparticles and bioparticles at single-particle resolution.

In this review, we touch on recent fundamental advances in using microelectromechanical systems and microfluidics chips in (i) magnetic nanoparticle synthesis; (ii) nanoparticle and microparticle manipulation and transport on a chip; as well as (iii) nanoparticle detection and magnetic characterization, with a focus on bioapplications. We discuss the fundamental goal of developing a “lab-on-a-chip” system to synthesize magnetic nanoparticles, label bioparticles, sort and detect them on a single chip. With this broad structure, here the goal is to, instead of including all available works, highlight advances in the field. We believe it will provide crucial information for researchers interested in lab-on-a-chip, microfluidics, magnetic nanoparticles, and their use in medicine and immunology.

## 2. Magnetic Materials 

All matter is “magnetic” (i.e., quantum exchange interactions between the electronic orbitals and spins in atoms exist); however, the materials are also classified based on their response to an external magnetic field [25,26,27]. Diamagnetic materials are the ones in which all electrons exist in a paired format, and there is no exchange interaction between the atomic magnetic moments. Thus, the net magnetic moment in diamagnetic materials is zero, which means they do not show magnetic properties in the absence of an external magnetic field. The diamagnetic materials repel the external field and get magnetized due to the additional angular momentum their electrons acquire. This behavior results in a negative slope in their susceptibility curve versus the externally applied magnetic field. Since all materials have electron pairs, they all show diamagnetic properties.

Similar to diamagnetic materials, in paramagnetic materials, no exchange interaction between atomic magnetic moments is present, and the net magnetic moment in the absence of the external magnetic field is zero. However, the unpaired electrons in these materials in an external magnetic field result in a net positive magnetic moment dominating the diamagnetic negative response due to their paired electrons.

In ferromagnetic materials (e.g., nickel, cobalt, and iron), permanent atomic magnetic dipoles are available even in the absence of an external magnetic field. These materials exhibit a strong negative exchange interaction which overcomes the diamagnetic behavior. They show a hysteresis behavior to the external magnetic field.

Antiferromagnetic materials show a solid positive exchange interaction. In these materials (e.g., chromium), below a critical temperature, called the Néel temperature (*T_N_*), application of a magnetic field aligns the adjacent atomic moments in an antiparallel format, which results in zero net magnetization. At higher temperatures, antiferromagnetic materials show paramagnetic behavior. In a special case of antiferromagnetic materials, called ferrimagnetic material (e.g., magnetite, Fe_3_O_4_, and maghemite, γ-Fe_2_O_3_) antiparallel moments form a non-zero net magnetic moment. Here we do not discuss the temperature behavior of the magnetic materials in detail.

Typically, small enough ferromagnetic nanomaterials with a single magnetic domain (i.e., nanoparticles with diameters as small as ~5–50 nm, depending on the material) are superparamagnetic materials. In these materials, the thermal fluctuations randomly flip the magnetization and eliminate the magnetic hysteresis behavior [28]. The magnetic susceptibility of superparamagnetic materials is higher than that of paramagnetic materials. An external magnetic field magnetizes superparamagnetic materials, and by increasing the magnetic field intensity, their magnetization increases up to their magnetization saturation point. However, by removing the magnetic field, they no longer show any magnetic interaction [29]. That means in the absence of the external magnetic fields, the superparamagnetic nanoparticles do not aggregate, which is an important property and makes them suitable for bio-applications. The total magnetic moment (*m_T_*) of superparamagnetic particles in classical electromagnetic is known as the Langevin function and is written as [26]

(1) $$m_{T}=Nm\left(\coth\left[\frac{m\left(\mu_{0}H\right)}{k_{B}T}\right]-\left[\frac{k_{B}T}{m\left(\mu_{0}H\right)}\right]\right)$$

where *m*, *N*, *T*, *µ*_0_, *H*, and *k_B_* stand for the magnetic moment per particle, number of particles with magnetic moment m, temperature, vacuum permeability, magnetic field intensity, and the Boltzmann constant, respectively. In quantum mechanics, the equation for superparamagnetic particles is expressed as [26]

(2) $$m_{T}=NgJm\left(\left[\frac{2J+1}{2J}\coth\left[\left\{\frac{2J+1}{2J}\right\}\frac{gJm\left(\mu_{0}H\right)}{k_{B}T}\right]\;-\frac{1}{2J}\coth\left[\left\{\frac{1}{2J}\right\}\frac{k_{B}T}{gJm\left(\mu_{0}H\right)}\right]\right]\right)$$

where *g* and *J* represent the spectroscopic splitting factor and the total angular moment, respectively. 

Available magnetic nanoparticles are usually the ferrites or iron oxide nanoparticles (e.g., maghemite γ-Fe_2_O_3_ or magnetite Fe_3_O_4_), metallic nanoparticles (e.g., Fe and Co), or alloy nanoparticles (e.g., Co/Pt alloys). These nanoparticles are also sometimes synthesized with a coating (e.g., for increasing biocompatibility).

## 3. Synthesis 

**Figure 1:**
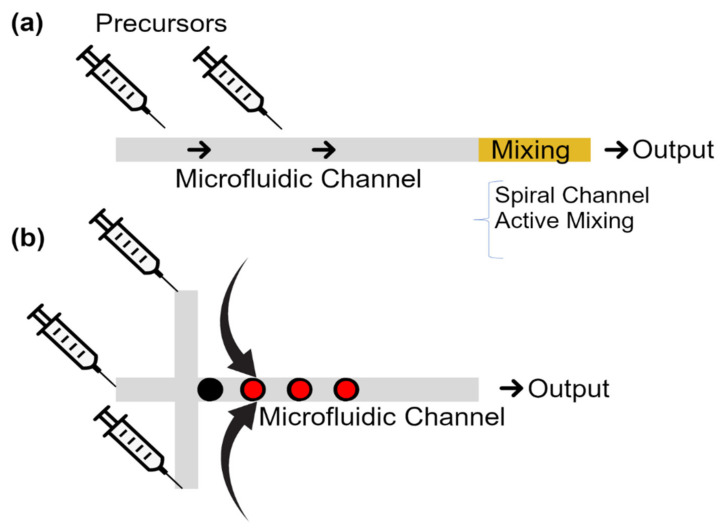
The two main methods of microfluidic-based nanoparticle synthesis. (**a**) Continuous-flow microreactors, where the particles are synthesized in a microchannel, and (**b**) the droplet-based microreactor, where the nanoparticles are synthesized in a droplet. The yellow area (mixing) in (**a**) is optional.

Edel and coworkers in 2002 proposed synthesizing nanoparticles in a microfluidic chip [30]. This method increases the control over key reaction parameters such as the temperature, reagent concentrations, flow rates, and reaction time. This ability results in better control over the particle characteristics, such as the particle size distribution. The microfluidic-based magnetic particle synthesis methods are the continuous-flow and droplet-based microreactors and will be reviewed here (see Figure 1). The concept of nanoparticle production in a continuous flow format lowers the possibility of coalescence of the synthesized nanoparticles. Moreover, the droplet reactors isolated from each other answer this need in a different way. We will also touch on the methods used for the synthesis of microparticles encapsulating magnetic nanoparticles.

### 3.1. Continuous-Flow Microreactors

The continuous-flow microreactors are the most commonly used microfluidic-based reactors for nanoparticle synthesis. As shown in Figure 1a, in this method, precursors get into a microfluidic channel, where the nanoparticles form. Because of the laminar flow in the microchannels, in this method, diffusion is the key mixing mechanism. The achieved slow mixing process guarantees reproducible controlled nanoparticle production. However, in the synthesis of some nanoparticles, faster interactions are needed and, thus, another mixing method (e.g., spiral channels or active mixing methods) is used.

**Figure 2:**
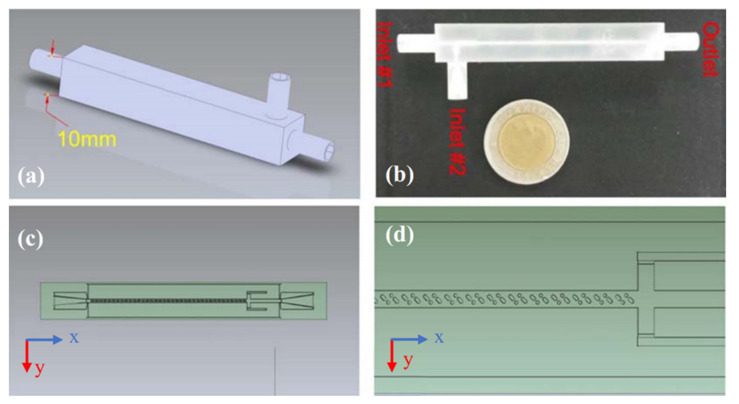
A 3D printed microfluidic chip for magnetic nanoparticle synthesis based on co-precipitation. (**a**) A 3D schematic of the model. (**b**) The 3D printed device. (**c**) The microfluidic channels. (**d**) The hurdles in the channel. Reprinted with permission from M. D. Aşık et al., 2021 Journal of Nanoparticle Research. 23 62 [31]. Copyright 2021 Springer Nature.

Co-precipitation is one of the main methods for synthesizing nanoparticles in wet chemistry. Magnetic nanoparticles are commonly synthesized by co-precipitation of iron salts with a base. In addition to the conventional co-precipitation in bulk chambers, microfluidic chips can also play the reaction chamber role in this technique. Figure 2, from [31], represents such a microfluidic chip for magnetic particle synthesis. This device is 3D printed, and in its design, as shown in Figure 2d, hurdles are included to affect the particle size.

Researchers have developed a spiral microfluidic device with the continuous flow to synthesize magnetic nanoparticles based on the co-precipitation method in its ~20 µm deep microchannels [32]. The spiral shape of the microfluidic channel in this design does the mixing step in the co-precipitation. In this technique, iron (i.e., the mixture of iron (II) and iron (III) acidic solutions) and base (i.e., 5 M NaOH solution) precursors enter into the main microfluidic channel, where the nanoparticles start to form and grow. The authors claimed that they have good control on the particle size and magnetic characteristics, and have used this device to synthesize ~10 nm magnetite nanoparticles with the magnetization of more than 50 emu/g. Moreover, a similar method was previously used for synthesizing cobalt nanoparticles [33,34].

Particle agglomeration during the synthesis is one of the main challenges. One solution to better overcome this problem is to maintain an intermolecular distance between the particles by providing a surfactant coating. A typical method is the use of long-chained polymers, such as dextran. Synthesis of such particles using a continuous-flow microreactor has been shown recently [35]. Iron sulfate/iron nitrate and NaOH/dextran from two microchannels came together. The authors claimed that they produced superparamagnetic spherical nanoparticles with ~10 nm size and magnetic saturation of 40–60 emu/g. Another research group has used a similar microfluidic chip for controlled nanoparticle synthesis and developing a core alloying and shell gradient doping strategy [36]. Their method shows good potential in the controlled synthesis and surface modification of magnetic nanoparticles. They put metal alloys inside the nanoparticles, as the nanoparticle core, and put a metal oxide on top, as the shell, forming particles smaller than 5 nm.

In order to better study and control the magnetic nanoparticle synthesis, researchers have presented a programmed microfluidic chip by which four stages, including mixing, nucleation, growth, and termination during the nanoparticle synthesis, could be observed separately [37]. This device allows researchers to optimize each stage by tuning the channel length, flow rate, concentrations, and temperatures and create nanoparticles as small as 5 nm. 

**Figure 3:**
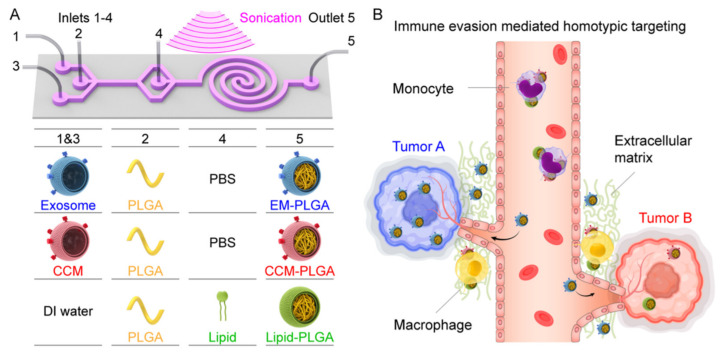
Schematic of the microfluidic sonication method proposed by [41] to assemble biomimetic core-shell nanoparticles. (**A**) The microfluidic chip uses sonication and hydrodynamic mixing methods for the synthesis of exosome membrane (EM)-, cancer cell membrane (CCM)-, and lipid-coated nanoparticles. The precursors are injected into the chip from the inlets (1–4), and the product is collected from the outlet. (**B**) Reduced uptake of the produced biomimetic nanoparticles by the peripheral blood monocytes and extracellular matrix macrophages is shown. Reprinted with permission from C. Liu, et al., 2019 Nano Letters 19(11), 7836–7844 [41]. Copyright 2019 American Chemical Society.

In many bioapplications, magnetic nanoparticles are in vivo tools. Thus, they need to escape the immune system. Microfluidic chips have also answered this need by synthesizing coated magnetic nanoparticles. For example, researchers have reported the microfluidic-based synthesis of ~6 nm iron oxide magnetic nanoparticles encapsulated in poly(methyl methacrylate) with a total size of 100–200 nm [38]. Furthermore, scientists have reported magnetic nanoparticles loaded with a drug and molecules specific to a target tissue. They mixed precursors in a continuous flow microfluidic chip [39]. However, microfluidic chips have also answered this need better by producing biomimetic magnetic nanoparticles. Researchers have used microfluidic electroporation-facilitated chips to put the Fe_3_O_4_ magnetic nanoparticles into red blood cell vesicles [40]. They claim the resulting magnetic nanoparticles show better treatment effects than the traditionally fabricated nanoparticles. Similarly, researchers have proposed a hybrid microfluidic sonication and hydrodynamic mixing approach to synthesize nanoparticles with an exosome membrane, which also has the potential for producing magnetic nanoparticles [41] (see Figure 3). 

### 3.2. Droplet-Based Microreactors

Although co-precipitation is a fast and low-cost method, channel clogging is one potential problem with the continuous flow (single-phase) co-precipitation-based microfluidic chips. This problem is even worse in magnetic nanoparticle synthesis because of the high reactivity of the magnetic precursors [42].

**Figure 4:**
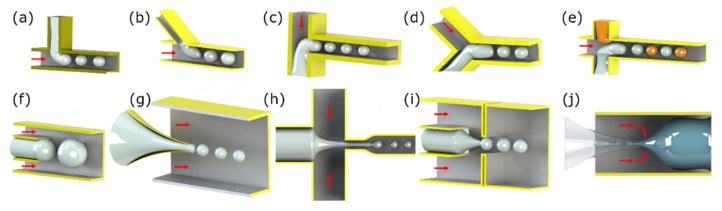
Schematics of various droplet-based microfluidic designs used in various works, including (**a**–**e**) the cross-flow, (**f**,**g**) the co-flow, and (**h**–**j**) the flow-focusing, are shown. Here the fluid in blue color stands for the magnetic materials used for creating the magnetic particles, and the red arrows depict the direction of the continuous phase flow. In (**e**), two dispersed phases form (blue and orange).

Droplet-based microfluidic is one of the widely used methods in producing both droplets and micro/nanoparticles, and a good solution to remarkably overcome the clogging challenge, cross-contamination, sample loss, long diffusion time, and the Taylor dispersion effect mostly seen in continuous-flow microreactors [43]. As shown in Figure 1b, precursors enter the microfluidic channel to form tiny droplets. Each droplet reacts as a tiny isolated reactor, in which the reaction of interest occurs. It is also possible to form particles with shells using a single-step reaction on a single chip. Various droplet-based microfluidic techniques are already developed, including (i) cross-flow [44,45,46,47]; (ii) co-flow [48,49]; and (iii) flow-focusing [50,51,52,53]. Figure 4 illustrates schematic of several droplet-based geometries.

In the cross-flow design (e.g., the T-junction, Y-junction, etc.) the channels transporting the dispersed phase (i.e., the precursors) intersect the main channel, which carries the continuous phase. At the junction, the droplets of dispersed phase form and transport in the continuous phase (see Figure 4a–e). As illustrated in Figure 4e, two sets of particles with different materials can also develop to merge afterward. In the co-flow geometry, the dispersed phase in the middle is symmetrically surrounded by the continuous phase, and both share the same flow direction in coaxial microchannels (see Figure 4f,g). In the flow-focusing configuration, the dispersed phase in the middle is symmetrically sheared by the continuous phase and pushed through an orifice (see Figure 4h–j). In the simplest form, the flow-focusing design consists of a continuous phase containing channel pair(s) on sides (see Figure 4h). In the micro-capillary-based 3D flow-focusing device, a glass micro-capillary forms the required orifice. Moreover, Figure 4j illustrates a configuration based on two glass micro-capillary and the combination of flow focusing and co-flow designs. 

Researchers have used droplet microfluidics for synthesizing magnetic nanoparticles too. They have used a type of capillary flow-focusing droplet-based microfluidic design to synthesize dextran-coated superparamagnetic iron oxide nanoparticles [42]. In this method, precursor streams of Fe^2+^/Fe^3+^/dextran and NH_4_OH entered into a stream of octadecene carrier fluid, and iron oxide nanoparticles were produced inside droplets. The authors claimed synthesis of crystalline particles with the magnetization of 58 emu/g and narrow size distribution with a mean diameter of 3.6 nm and standard deviation of 0.8 nm. This span is a narrower range compared to earlier reports [54]. 

**Figure 5:**
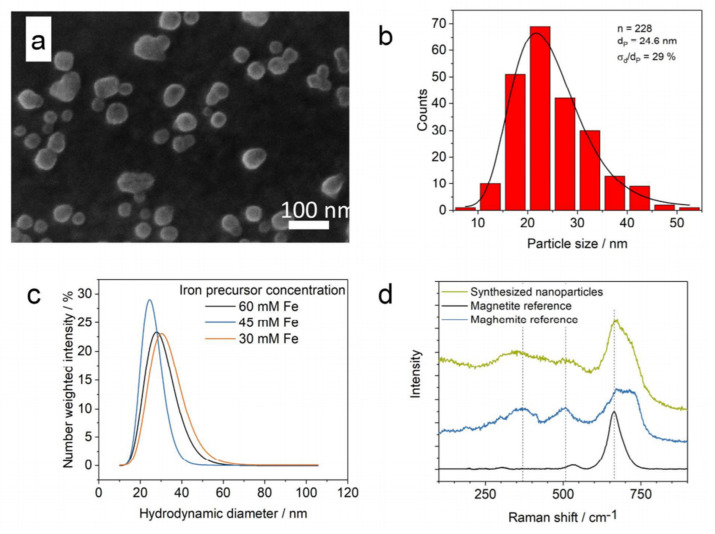
(**a**) Field emission scanning electron microscopy (FESEM) image of the magnetic iron oxide nanoparticles synthesized in [55]. (**b**) The particle size distribution. Here n, d_p_, and σ_p_/d_p_ stand for the particle quantity, particle diameter, and the relative standard deviation at the lowest base concentration, respectively. (**c**) Number weighted intensity versus the hydrodynamic diameters, measured by dynamic light scattering (DLS), is plotted. (**d**) Raman spectra of the nanoparticles are illustrated. Reprinted with permission from J. Bemetz, et al., 2018 Analytical Chemistry 90(16), 9975–9982. [55]. Copyright 2018 American Chemical Society.

Dr. Seidel and his coworkers synthesized magnetic nanoparticles by co-precipitation of ferric- and ferrous chloride with sodium hydroxide in a 3D flow-focusing microfluidic chip [55]. In this method, the iron salt precursor stream, produced by dissolving iron(II) chloride and iron(III) chloride with a molar ratio of 1:2, was flown into the base. Figure 5, which is taken from [55], shows their synthesized nanoparticles.

Researchers have also used the flow-focusing approach in a recent work to synthesize magnetic core/chitosan shell nanoparticles [56]. These nanoparticles carry a drug (e.g., cisplatin) to be released using a crosslinker (e.g., tripolyphosphate). They synthesized these particles using an integrated microfluidic device composed of three stages. First, they used precipitation to produce a superparamagnetic core. Then, during the encapsulation stage, a polymer shell formed on the magnetic core. Finally, they stabilized the core-shell particles and loaded them with the drug. The authors in this work estimated the time required for mixing by Equation (3):

(3) $$t=\frac{w_{f}{}^{2}}{4D}=\frac{w^{2}}{9D}\frac{1}{{\left(1+\frac{1}{FRR}\right)}^{2}}$$

where *w_f_*, *D*, *w*, and *FRR* represent the inner stream width, the solvent diffusion constant, the channel width, and the flow rate ratio of the focused stream to the adjacent streams, respectively [56].

Frenz and coworkers have presented a method to create droplet pairs for precipitating iron oxide nanoparticles by fusion droplets containing required reagents [57]. This method, which is based on a cross-flow design, provides good control on the synthesis parameters. Electrocoalescence mixes the reagents in the droplet pairs. The authors claim that ~2 ms after mixing the droplets, the nanoparticles with a diameter of ~4 nm are formed. The idea of producing droplets based on a cross-flow microfluidic design followed by droplet mixing is also recently used to create chitosan-coated iron-oxide nanoparticles [58]. The microfluidic chip in this work is based on the design shown in Figure 4e. In this work, as opposed to electrocoalescence, spiral microfluidic channels merge the droplet pairs and mix chitosan and iron chloride droplets.

In Table 1, examples of microfluidic-based magnetic nanoparticle synthesizes are listed.

### 3.3. Microspheres Encapsulating Magnetic Nanoparticles

In addition to synthesizing magnetic nanoparticles, microfluidic chips can magnetically label microparticles. A problem in drug delivery is the fast clearance of the nanoparticles in the body. Thus, targeted nano-drug delivery to cancer cells has a great promise for enhancing their cellular uptake. Using microfluidic chips, researchers have developed various types of drug-releasing particles, some good reviews on which are available [64]. For instance, scientists have developed pH-responsive microparticles with a great potential for targeted drug delivery. They fabricated the polymer/porous silicon composite microparticles loaded with multiple drugs in a flow-focusing capillary device [65]. The particles remained unchanged at pH from 1.2 to 5.5; however, they started to dissolve and released ~50% of their drugs in two hours between pH 6.0 and 6.5. At pH above 6.5, the particle completely collapsed and released all of its loaded drugs. In another study, scientists used the same particles for encapsulating drug-loaded silicon nanoparticles and magnetic bacterial iron oxide nanowires [66].

Multi-stimuli-responsive particles enhance the controlled drug release profile [67]. The drugs can be released using temperature and/or pH change. Researchers have used a capillary-based droplet microfluidic to produce these particles encapsulating magnetic nanoparticles. The magnetic nanoparticles move toward the desired sites under an external magnetic field and give the capsules site-specificity properties.

**Figure 6:**
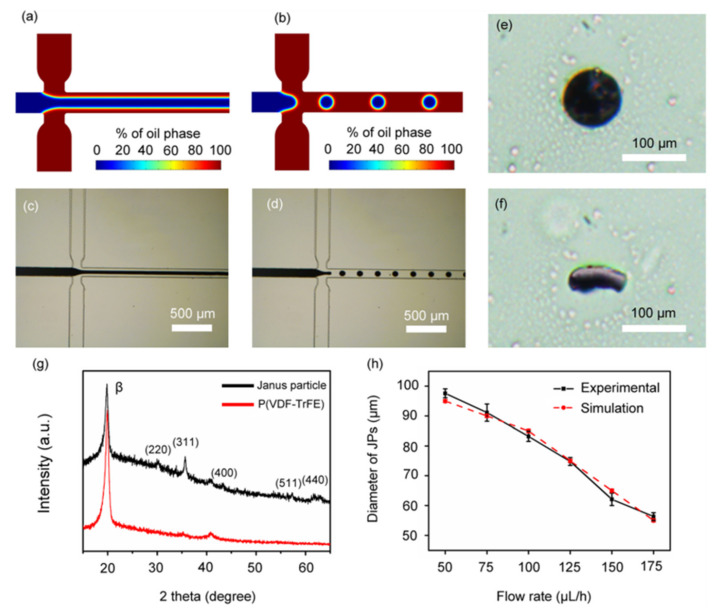
(**a**–**d**) Simulation and experimental images of the laminar flow and the microfluidic device. (**e**,**f**) Sample images of a disk-like Janus particle. (**g**) XRD analysis of the synthesized particles. (**h**) Simulation and experimental results for the particle diameters at various flow rates. Reprinted with permission from X. Yu, et al., 2016 Applied Physics Letters 108, 073504 [72]. Copyright 2016 AIP Publishing.

Researchers have also reported the non-spherical hydrogel microparticles encapsulating magnetic nanoparticles [68]. They generated the droplets in a T-junction-based droplet microfluidic chip and gave them time to relax and take the shape of the confining microchannel. Then, they used UV photopolymerization to fix the particle shapes. The microfluidic channels let researchers ensure achieving uniform UV energy and particle geometry distribution. In another work, scientists synthesized disk-like magnetic Janus particles. Figure 6 shows the microfluidic design used in this work, a sample of the synthesized particle, and the related tests. The authors use the X-ray powder diffraction (XRD) analysis to confirm the regular crystal structure of the poly(vinylidene fluoride-trifluoroethylene) (P(VDF-TrFE)) and Fe_3_O_4_ nanoparticles. They also simulated the size of the synthesized Janus particles at various flow conditions (see Figure 6h).

Droplet microfluidics is widely used in single-cell RNA-seq to extract the genomic data of the cells at the single-cell level [69,70,71]. In this technique, microdroplets encapsulate a single barcode-carrying magnetic bead together with a single cell. After cell lysis, the magnetic particles collect the RNAs of the cells to be further processed and studied.

Table 2 tabulates the advantages and challenges associated with the conventional and microfluidic reactors for synthesizing magnetic nanoparticles.

## 4. Particle Manipulation

Manipulating magnetic nanoparticles, including sorting and separating them from a mixture, is one of the main goals in the field of lab-on-a-chip. Microfluidic chips provide great opportunities for manipulating microparticles as well as nanoparticles. There are multiple methods for microfluidic-based nanoparticle sorting, including the ones based on hydrodynamic [74,75,76], dielectrophoretic [77,78], optical [79,80,81], acoustic [82,83], and magnetic forces. Here, we focus on the methods based on magnetic forces specially designed for manipulating magnetic particles.

There are three magnetic nanoparticle manipulation microfluidic types, including (i) the ones equipped with external coils or permanent magnets(s); (ii) the ones equipped with magnetic micro-wires and micro-coils; and (iii) the ones with embedded magnetic thin films. Scientists have shown numerical analysis results of the transport of magnetic nanoparticles in a microfluidic chip exposed to an external magnetic field [84,85]. The magnetic force on magnetic particles can be written as Equation (4):

(4) $$\vec{F}=\left(\vec{m}.\nabla \right)\vec{B}$$

where *m* and *B* stand for the magnetic dipole moment and the magnetic flux density, respectively. Superparamagnetic materials in an external magnetic field, weaker than their saturation point, show a magnetization given by:

(5) $$\vec{m}=\left(\chi_{p}-\chi_{f}\right)V_{p}\vec{H}$$

where *χ_p_*, *χ_f_*, *V_p_*, and *H* are the magnetic susceptibility of the magnetic particle, the magnetic susceptibility of the surrounding fluid, the particle volume, and the magnetic field intensity, respectively. One can approximate the superparamagnetic nanoparticles, and the magnetically labeled particles (e.g., the cells), with magnetic dipoles with magnetization expressed in Equation (5). Thus, the magnetic field intensity and the field gradient play important roles in defining the magnetic force acting on the particles. The magnetic field can be written as

(6) $$\vec{H}\left(\vec{r}\right)={\vec{H}}_{ext}+{\vec{H}}_{mag}\left(\vec{r}\right)+{\vec{H}}_{wire}\left(\vec{r}\right)$$

where *H_ext_*, *H_mag_*, and *H_wire_* stand for the applied external magnetic field, the magnetic field produced by the magnetized magnetic materials in the systems, and the magnetic field produced by current-carrying wires in the system, respectively. Each term in Equation (6) may or may not exist in various applications. *H_mag_* can be calculated by

(7) $${\vec{H}}_{mag}\left(\vec{r}\right)=-\nabla \varphi_{m}\left(\vec{r}\right)$$

where *ϕ_m_* is the magnetic potential and can be derive as

(8) $$\varphi_{m}\left(\vec{r}\right)=\frac{1}{4\pi }∯\frac{\sigma \left({\vec{r}}_{s}\right)}{\left|\vec{r}-{\vec{r}}_{s}\right|}ds$$

where |*r* − *r_s_*| represents the distance between the observation point, *r*, and the source point, *r_s_*. In this equation, *σ* is the equivalent magnetic charge density on magnetic materials (e.g., thin films) when exposed to an external magnetic field and is calculated by

(9) $$\sigma =\left({\vec{H}}_{in}-{\vec{H}}_{out}\right).\hat{n}$$

where *H_in_* and *H_out_* are the magnetic fields inside and outside magnetic materials, respectively, and n stands for the local outward unit vector normal to the surface.

*H_wire_* in Equation (6) can be derived using the Biot–Savart law. Assuming the current-carrying thin films (in the case of microfluidics) produce sheet current, *K*(*r_s_*), the generated magnetic field can be calculated as

(10) $${\vec{H}}_{wire}\left(\vec{r}\right)=\frac{1}{4\pi }∯\frac{\vec{K}\left({\vec{r}}_{s}\right)\times \left(\vec{r}-{\vec{r}}_{s}\right)}{{\left|\vec{r}-{\vec{r}}_{s}\right|}^{3}}ds$$

### 4.1. Particle Manipulation with an External Permanent Magnet or Electromagnet

Particle manipulation with a permanent magnet is one of the simplest widely used methods in both conventional and microfluidic-based particle separations. Scientists have reported that a simple microfluidic chip equipped with a permanent magnet can separate magnetic nanoparticles based on their size [86]. This technique uses the applied magnetophoretic forces on the nanoparticles moving inside the microchannels in a laminar flow. The authors claim that by adjusting the distance between the magnet and the microchannel, they have successfully separated Fe_3_O_4_ nanoparticles of 40 to 280 nm into two fluids with mean diameters of 90 and 160 nm. Other researchers have also used similar methods [87].

Scientists have proposed a diagnostic assay for the detection of methicillin-resistant Staphylococcus aureus (MRSA) by extraction and amplification of nucleic acids using magnetic particles inside a microfluidic chip [88]. They used magnetic beads conjugated with probes specific to the target deoxyribonucleic acid (DNA) and a permanent magnet to collect the DNA inside the microfluidic chips.

**Figure 7:**
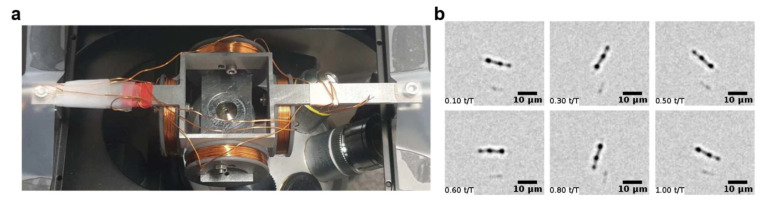
(**a**) The experimental setup for the magnetic mixing system proposed in [93]. (**b**) Snapshots of a rotating magnetic nanobar in a microdroplet in an external rotating magnetic field. Reprinted with permission from P. Gires, et al., 2020. Scientific Reports 10, 10911 [93] under Creative Commons Attribution 4.0 International License http://creativecommons.org/licenses/by/4.0/, accessed on 28 June 2021. Copyright 2020 Springer Nature Publishing.

Microfluidic magnetic mixing is another application of manipulating magnetic nanoparticles in microfluidic chips [89,90]. It is shown that the application of an external magnetic field to the microfluidic chip loaded with nanoparticles results in forming nanoparticle chains. Now, by rotating the external field, the nanoparticle chains rotate and mix the surrounding fluid. The created mobile swarms can deform and perform controlled splitting and merging [91]. The assembled chains may have applications in targeted delivery, maskless ribbon-like patterning for microfabrication, and micromanipulation. Some researchers, as opposed to nanoparticle chains, have used nanorods [92]. This technique has also been used for generating vortexes inside microdroplets in microfluidic chips [93]. The authors have reported magnetic nanobars with diameters of ∼300 nm and lengths in the range of 100 nm up to several micrometers. Figure 7 illustrates the experimental setup, in which the chip is placed in between four coils, and images of the mixing magnetic nanobars.

### 4.2. Particle Manipulation with Embedded Micro-Wires and Micro-Coils 

**Figure 8:**
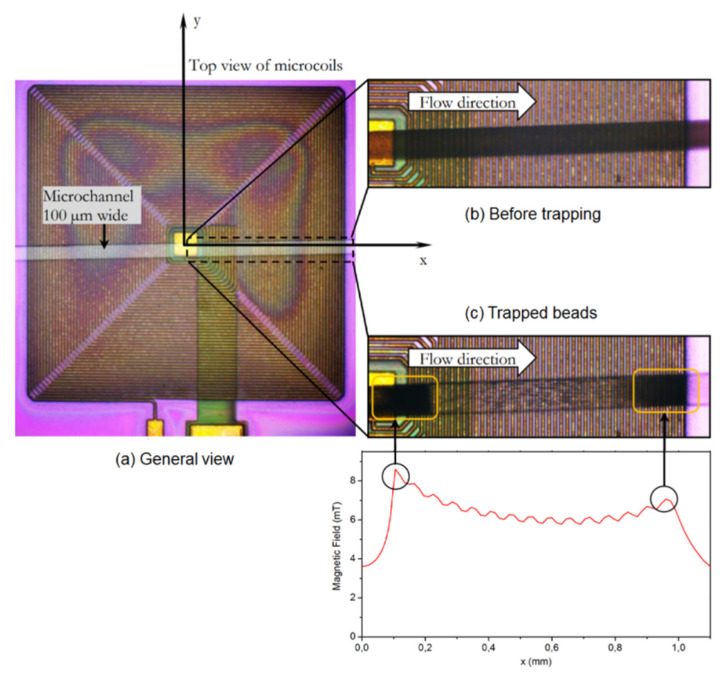
Magnetic nanoparticles trapped by micro-coils in a microfluidic device are illustrated. (**a**) The whole chip. (**b**) Injection of particles. (**c**) Beads trapped with coils. Reprinted with permission from O. Lefebvre, et al., 2020 Micromachines 11(3), 257 [96] under the terms and conditions of the Creative Commons Attribution (CC BY) license http://creativecommons.org/licenses/by/4.0/, accessed on 28 June 2021. Copyright 2020 MDPI.

Current-carrying wires produce magnetic fields, and this is the basic idea behind microfluidics in which wires and coils provide the force required for manipulating magnetic nanoparticles. Researchers have used this technique widely [94,95]. Figure 8, taken from [96], shows how the micro-coils attract the magnetic nanoparticles in a microfluidic channel. Researchers have integrated micro-coils in a microfluidic chip for trapping and sensing the barcode-carrying magnetic nanoparticles [96,97]. This chip works an enzyme-linked immunosorbent assays (ELISA)-based immunoassay. The authors have used the developed chip to detect ovalbumin with the ability to detect protein concentrations as small as ~10 pg/mL.

Scientists have reported a microfluidic chip in which giant magneto-resistance (GMR) sensors are combined with current-carrying micro-wires [98]. In this method, a direct current (DC) is applied to the micro-wires to produce the required magnetic field and collect magnetic nanoparticles (i.e., markers). They claim detection of particle concentrations of ~500 pg/mL, quantifying them in a linear scale, and reading the sensor voltage for a collection of nearly 20 magnetic nanoparticles. In a similar work, researchers fabricated micro-wires in the shape of a coil on a chip to cover the wafer surface around a magnetoresistive sensor [99]. These micro-coils produce a magnetic field to accumulate the magnetic particles on the sensor area. The authors have shown this ability by both using a numerical method and running the required experiments. 

Since, in some designs, the micro-coils are far away from the magnetic nanoparticles, large electric currents are needed to produce strong enough magnetic fields. Thus, in such chips, a heat management system is required to dissipate the produced resistive heat. Scientists have answered this need by employing copper sheets [100]. Using this chip, simultaneous attraction and repulsion of 300–500 nm magnetic nanoparticles is achieved, an ability which, as authors claim, results in particle concentration enhancement. 

**Figure 9:**
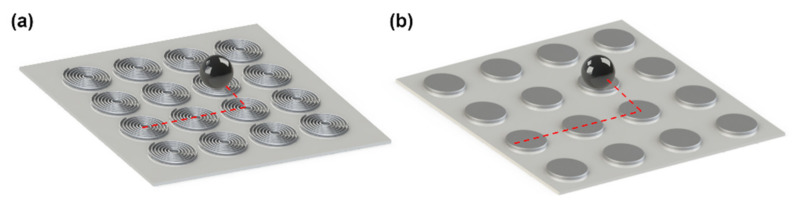
Schematics of magnetic single-particle transport using an array of (**a**) coils and (**b**) magnetic thin disks. The red dashed lines show the movement of the particle (black circle) from one coil/disk to the other one.

Forming an array of particles and manipulating single particles are two important examples of the main goals in the lab on a chip system, with applications in various fields, including single-cell biology. To achieve these aims, we can fabricate an array of individually-triggered micro-coils on chips. Figure 9a shows this architecture. By sequential triggering the micro-coils, one can define the single-particle trajectories. However, in large arrays, this wiring system becomes complicated. Moreover, resistive heating due to the current-carrying coils can be challenging. To answer this problem, as opposed to using active coils, it is possible to magnetize magnetic thin films fabricated on a chip, as shown in Figure 9b. As it will be explained in the next section, in this method no external current is needed, which is a key advantage. In Figure 9, sample particle trajectories show how the coils and magnets move single particles in the two methods. 

### 4.3. Particle Manipulation with Embedded Magnetic Thin Films

Researchers have used an array of magnetic micro-strips to transport magnetic particles [101]. A vertical field and an in-plane field magnetize the magnetic micro-strips. They have shown that using a proper sequence of applied external magnetic fields, the energy minima move from one strip to another and thus transports the magnetic particles. They have demonstrated that the particles can move at various speeds based on their size. Hence, in addition to transporting particles, this method works as a size separation technique. 

Scientists have proposed a microfluidic platform with zig-zag-shaped magnetic structures for manipulating magnetic particles [102]. By applying external magnetic fields in the required direction, magnetic energy wells move to the nearest corner. Thus, the following magnetic particles move towards the magnetic track. Moreover, it is shown that circular magnetic patterns can separate particles based on their size [103]. 

Recently, we have introduced magnetophoretic circuits, for precise manipulating magnetic particles and magnetically labeled cells with magnetic nanoparticles [104,105,106,107,108], at single-particle resolution. The proposed microfluidic chips are composed of overlaid magnetic nano-films (e.g., permalloy), and current-carrying ~100 nm thick micro-wires. The circuits consist of circuit elements such as conductors, diodes, capacitors, and transistors. The microfluidic chip is exposed to an external rotating magnetic field which produces the driving force for the magnetic particles. In passive circuit elements, all the particles are synced with the external magnetic field. Thus, a large number of individual particles move in parallel. However, when required, applying an appropriate electric current to the gate of the magnetic transistors switches the trajectory of individual particles. 

**Figure 10:**
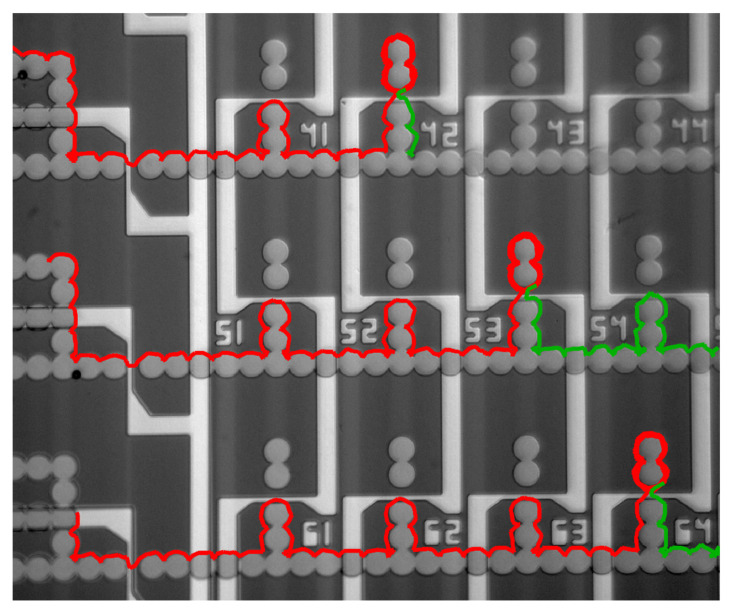
A small section of a magnetophoretic-based circuit designed and built for importing, storing, and exporting magnetic particles and magnetically labeled cells is shown. Here, three magnetic particles are temporarily stored in addressable sites 42, 53, and 64 (trajectories shown in red). At later times, the particles are exported from the chip (trajectories shown in green). Reprinted with permission from R. Abedini-Nassab, et al., 2015 Advanced Materials 27, 6176–6180 [105]. Copyright 2015 John Wiley and Sons.

We can combine these elements to design circuits for the precise manipulation of magnetic particles with special applications. For example, we designed a random access memory, similar to memory chips in computers, with the same memory architecture [105]. Thousands of magnetically labeled cells and particles can be assembled into addressable memory cells (i.e., storage sites) for dynamic studies [109,110] (see Figure 10). Moreover, by addressing specific storage sites, one can extract the particles of interest from their storage sites for further off-chip analysis (e.g., single-cell next-generation sequencing). Other groups have also shown that magnetophoretic circuits can deliver protein-functionalized particles and cells and store them in individual apartments [111]. In another work, similar technology is used for magnetic particle disaggregation [112]. Researchers have used this tool to detach the aggregated particles and form single particles, which move along the magnetic tracks. 

**Figure 11:**
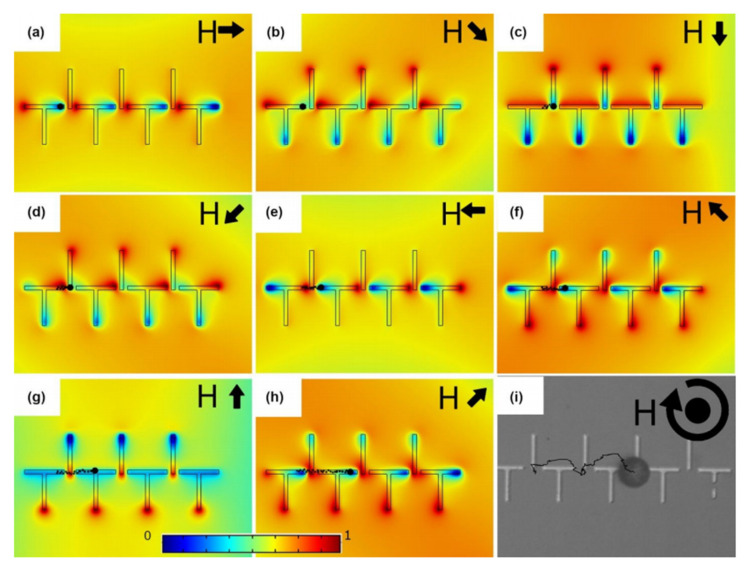
(**a**–**h**) Magnetic energy distribution simulation results for the magnetic thin film pattern exposed to a 3D rotating magnetic field with a cone angle of 45 degrees are shown. The black arrows stand for the in-plane magnetic field direction in each panel. The black circle depicts the particle, the trajectory of which is illustrated by the dotted line. The blue and red areas stand for the energy minimum and maximum, respectively. (**i**) The experimental trajectory of a magnetic particle transported along the magnetic track is shown with the black line. Reprinted from R. Abedini-Nassab, et al., 2021 Lab on a Chip 21, 1998–2007 [115] with permission from Royal Society of Chemistry.

Furthermore, to prevent particle-particle attractive force in an in-plane field, we have demonstrated magnetophoretic circuits operating in a three-dimensional external magnetic field [113,114,115]. The vertical magnetic field bias in this platform results in particle-particle repulsion force and prevents particle cluster formation, which may be seen in two-dimensional fields. Figure 11 illustrates the simulation results for the energy distribution which indicates how the particle of interest moves along the magnetic tracks from its initial position in Figure 11a to its position in Figure 11h in a single period. By further rotating the magnetic field, the particle moves further, the experimental trajectory of which is illustrated in Figure 11i.

**Figure 12:**
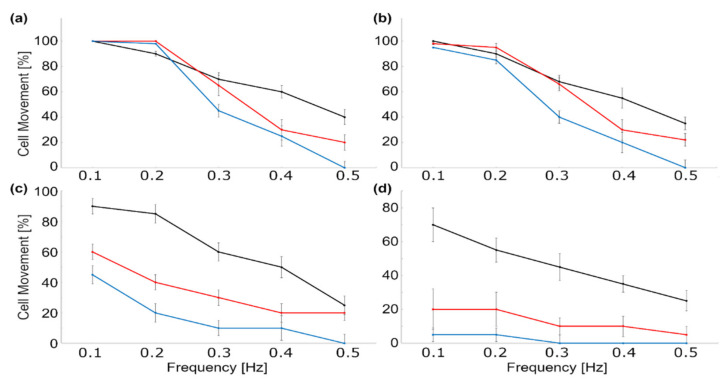
The experimental results showing the percentage of the AML (black and blue) and T cells (red) labeled with three different magnetic particles (see text for the details) moving on magnetic tracks in magnetophoretic circuits (**a**) 0, (**b**) 6, (**c**) 24, and (**d**) 48 h after magnetic labeling.

Table 3 lists the advantages and disadvantages of various magnetic particle manipulation methods. However, a general drawback of magnetic manipulation of magnetically labeled biological particles is the loss of magnetic nanoparticles over time. This phenomenon becomes problematic in applications in which one needs to manipulate particles a while after labeling them (e.g., dynamic study of single cells). In particular, we studied the transport of magnetically labeled cells using magnetophoretic circuits in multiple time points after cell labeling. Figure 12 illustrates the cell movement performance based on the applied magnetic field frequency for three different labeled cells. Based on the curves in this figure, at higher time points fewer cells move, indicating the magnetic nanoparticle loss over time. In these experiments, MOLM-13 acute monocytic leukemia (AML) cell line, magnetically labeled with antibody-conjugated magnetic nanoparticles (STEMCELL Technologies, Vancouver, Canada), human CD4+ T-cells labeled with anti-CD4 antibody labeled magnetic nanoparticles (STEMCELL Technologies, Vancouver, Canada), and THP-1 (ATCC TIB-202D) AML cells labeled with HLA-A2 conjugated particles were transported on magnetophoretic chips, the results of which are shown with black, red, and blue curves, respectively, in Figure 12. Although better cell handling techniques may answer the problem in our study, this challenge exists and needs to be answered in the future by synthesizing magnetic nanoparticles, which can stay inside cells or on their membrane for a longer time.

## 5. Detection and Characterization

Magnetic sensors can detect and characterize magnetic nanoparticles in microfluidic chips. Magnetic field sensors and detectors are widely used in various applications, including industrial navigation sensors [116,117,118], storage technologies [119,120,121], and biosensors [122,123,124]. Various types of magnetic sensors, including superconducting quantum interference devices (SQUIDs), magnetoelectric sensors, anisotropic/giant/tunneling magnetoresistive sensors, magnetorelaxometry-based sensors, optically pumped sensors, Hall effect sensors, and so on are available [125]. Here we review the recent and important works on anisotropic/giant/tunneling magnetoresistive sensors, magnetorelaxometry-based sensors, and some other innovative microfluidic-based sensors. The magnetoresistive (MR) effect refers to the electrical resistance change of the sensor due to the change of the applied magnetic field. The integration of MR sensors with microfluidic chips reduces the distance between the magnetic nanoparticles on the sensor, lowers the preparation time (e.g., in bioassays), and increases the sensor sensitivity [19].

### 5.1. Anisotropic Magnetoresistive Sensor

**Figure 13:**
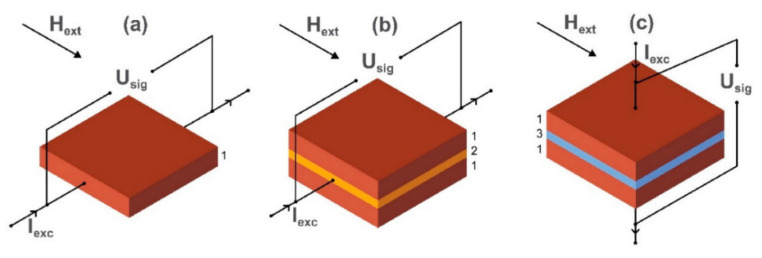
(**a**) Schematic of (**a**) anisotropic magnetoresistance, (**b**) giant magnetoresistance, and (**c**) tun-nel-ing magnetoresistance magnetic field sensors are shown. Here, Iexc, Hext, and Usig are the current flowing through the structure, the external magnetic field, and the applied voltage, re-spectively. Reprinted with permission from D. Murzin, et al., 2020 MDPI under the terms and conditions of the Creative Commons Attribution (CC BY) license http://creativecommons.org/licenses/by/4.0/, accessed on 28 June 2021.

One of the oldest available magnetometer methods is the one based on the anisotropic magnetoresistive (AMR) effect. The AMR effect is a property of some magnetic materials when the change between their magnetization orientation and the direction of the electric current affects their electric resistivity [119]. In the simplest form, the resistivity *ρ*(*ϕ*) can be written as Equation (11) [126],

(11) $$\rho \left(\varphi \right)=\rho_{⊥}+\left(\rho_{\left|\right|}-\rho_{⊥}\right)\cos^{2}\varphi$$

where *ϕ*, *ρ*_⊥_, and *ρ*_||_ stand for the angle between the electrical current and the magnetization direction, the resistivity for *ϕ* = 0°, and the resistivity for *ϕ* = 90°, respectively. A schematic for an AMR sensor is illustrated in Figure 13a.

**Figure 14:**
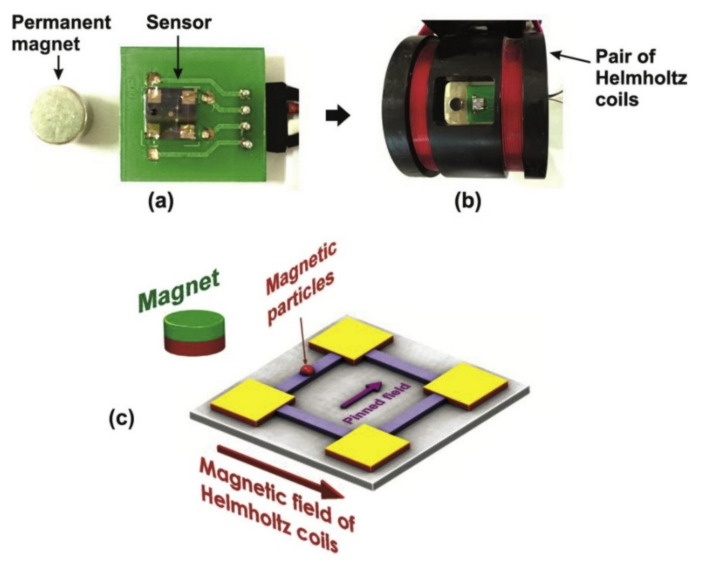
The experimental setup for the AMR magnetic particle detector in the Wheatstone bridge configuration is shown. (**a**) The AMR Wheatstone bridge device, (**b**) The chip in Helmholtz coils, and (**c**) The magnetic field components. Reprinted with permission from L.K. Quynh et al., 2016 Journal of Science: Advanced Materials and Devices 1(1), 98–102 [127]. Copyright 2016 Elsevier, under the terms of the Creative Commons CC-BY license http://creativecommons.org/licenses/by/4.0/, accessed on 28 June 2021.

Researchers have developed a serially connected ensemble of simple AMR elements of Ni^80^Fe^20^ film in Wheatstone bridge configuration to detect magnetic nanoparticles [127]. They claim that the proposed sensor shows a sensitivity of 2.15 mV/Oe. Figure 14 shows the chip that sits in Helmholtz coils.

Measurement of magnetic nanoparticles and detecting them has crucial applications in various fields such including biology. For instance, the same group has developed a disposable AMR-based chip based on the same technology to detect DNA labeled with magnetic beads [128]. They hybridized a magnetically labeled single-stranded target DNA with a specific DNA probe. They have reported a relatively linear sensor response in the target DNA range of 4.5 to 18 pmol. The authors claim their chip demonstrates higher efficiency and more cost-efficiency compared to conventional biosensors.

It is shown that fabricating multilayer AMR sensors (i.e., Ta/FeMn/[NiFe/FeMn]n/Ta), provides the opportunity to tune its detection over a wide range (i.e., 20.5 Gs to 116 G) [129]. This goal is achieved by fixing the thickness of the magnetic NiFe layer and choosing various numbers of NiFe/FeMn layers. Moreover, researchers have enhanced the voltage response of the AMR sensors in a Wheatstone bridge combination by using some resistor NiFe strips connected in series in the bridge arms. The authors agree that this connection results in higher thermal noise contribution. They claim a series-parallel configuration would have the best general results with reduced magnetic coercive field and the thermal noise contribution as well as a sensitivity 1.72 times higher than the one of the sensors with connections in series. They have used the proposed sensor to detect magnetic nanoparticles with a diameter of 50 nm, with a magnetic moment detection limit of ~0.56 µemu.

### 5.2. Giant Magnetoresistive Sensors

The giant magnetoresistive (GMR) effect is based on spin-dependent electron scattering and exists in metallic multilayer structures composed of alternating ferromagnetic and nonmagnetic layers (see Figure 13b). In a magnetic field, the magnetization directions of the two adjacent ferromagnetic layers can be either parallel or antiparallel. When the direction of magnetization in the ferromagnetic layers switches from antiparallel to parallel or wise versa, the electrical resistance of the structure changes [119,130]. There are some old highly cited works done in this field [131]; however, recently, the technology has attracted attention in various industries and the field of biosensing. In the GMR-based biochips, the GMR sensor detects magnetic nanoparticle tags of the biomolecules of interest. 

Researchers have developed a sensitive GMR-based sensor with NiFe/Cu/NiFe/Cu/Cr films for detecting magnetic particles [132]. The authors have reported large resistance variations caused by magnetic particles in the frequency range of 30 MHz∼120 MHz. A single sensor can detect the existence of a low number of particles. Another group has developed a wash-free magnetic bioassay based on GMR sensors [133]. They have used this chip for detecting the Influenza A virus in swine nasal swab samples. To perform the bioassay, they mixed the biotinylated Influenza A virus detection antibody (MAB8257B, EMD Millipore Corporation, Temecula, CA, USA, a mouse anti-influenza A monoclonal antibody specific for Influenza A virus nucleoprotein) with magnetic particles and the biological sample. After protein capture, they transferred the mixture to the GMR biochip. In this chip, the capture antibody available on the chip surface captures the target analyte-detection antibody-magnetic bead complex and forms a sandwich structure, producing the signal.

**Figure 15:**
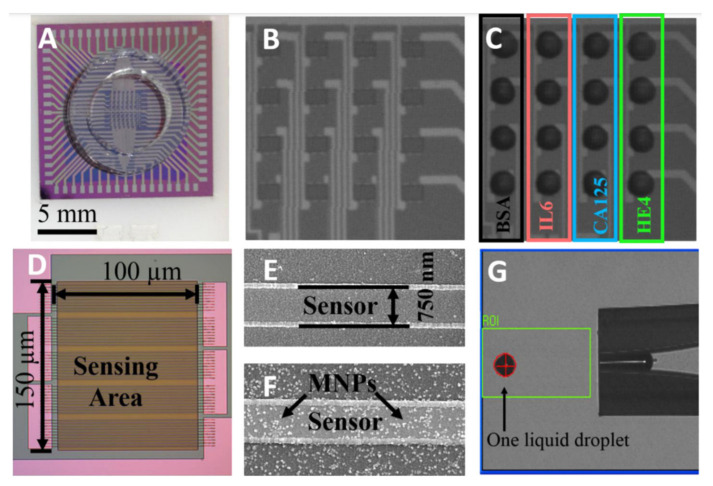
(**A**) The proposed GMR chip in [134]. The sensor array (**B**) before and (**C**) and after putting the samples on the chip. (**D**) A zoomed view of the sensors. SEM images of one GMR sensor strip (**E**) before and (**F**) after magnetic nanoparticles bound to its surface. (**G**) A piezo dispense capillary is used to dispense nanoparticles. Reprinted with permission from T. Klein, et al., 2019. Biosensors and Bioelectronics 126, 301-307 [134]. Copyright 2020 Elsevier.

**Figure 16:**
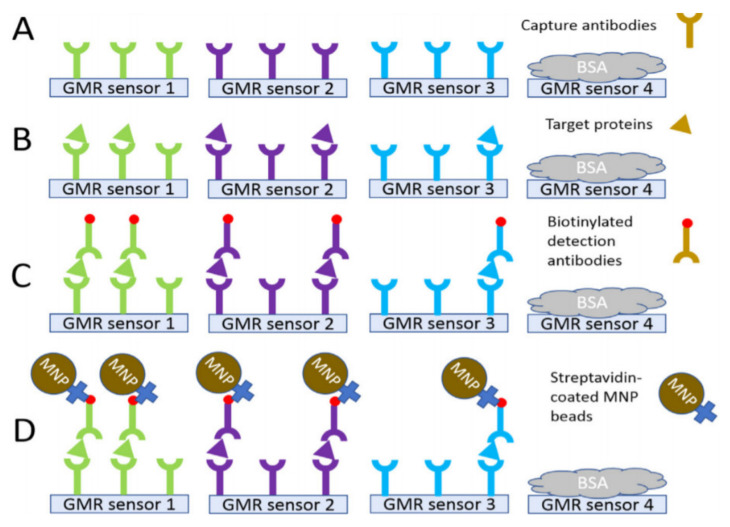
The Assay sequence proposed in [134] for detecting multiple magnetic nanoparticles carrying analytes. (**A**) Four groups of GMR sensors with different capture antibodies. (**B**) Samples with target proteins were introduced to the chips. (**C**) Biotinylated detection antibodies were introduced to the chip to selectively bind to the specific target analyte. (**D**) Streptavidin-coated magnetic nanoparticles were added to the chip surface, and GMR signals were monitored. Reprinted with permission from T. Klein, et al., 2019 Biosensors and Bioelectronics 126, 301–307 [134]. Copyright 2020 Elsevier.

Researchers at the University of Minnesota have demonstrated a GMR biosensor to detect ovarian cancer biomarkers [134]. Figure 15 illustrates pictures of the chip. The authors claim the proposed platform, which has features such as a mobile phone application and USB/Bluetooth communication, detects many protein biomarkers of human diseases. GMR arrays, each having the required capture antibodies, allowed the detection of multiple markers (see Figure 16). They have used Ademtech 200 nm magnetic beads, each composed of ~1000 magnetic nanoparticles with an average magnetic moment of ~2.3 × 10–16 emu. The authors mention that a potential drawback of using these large beads is the possibility of non-specific signals. To overcome this problem, they have included a negative control group to exclude the background signal. The authors claim detection of cancer antigen 125 (CA125 II), human epididymis protein 4 (HE4), and interleukin 6 (IL6), with limits of detections of 3.7 U/mL, 7.4 pg/mL, and 7.4 pg/mL, respectively.

Scientists at Stanford University used a similar method for the early detection of multiple biomarkers of cirrhosis [135]. The authors used the GMR-based chip for detecting intercellular adhesion molecule-1 (sICAM-1) and mac-2 binding protein glycosylation isomer (M2BPGi). They claim that the diagnostic performance of their tool is higher than the performance of the available clinical methods. 

Moreover, researchers have detected droplets created by a Y-shaped droplet microfluidic, using GMR sensors [136]. The droplets contained iron oxide magnetic nanoparticles with a diameter of 20 nm at a molar concentration of 230 mmol/L. The authors have shown that the in-flow detection of the mentioned nanoparticles for concentrations as small as 5.47 × 10^−9^ mol is possible. Another group has used a similar method for detecting and analyzing magnetic droplets generated by a T-junction microfluidic chip [137].

### 5.3. Tunneling Magnetoresistive Sensors

In a tunneling magnetoresistive (TMR) sensor, a thin insulating layer separates the ferromagnetic layers. In this device, a tunneling current passes through the insulating layer (see Figure 13c). These sensors with very high MR ratios have recently been emerging in various applications [138]. In TMR biosensors, by monitoring the change in resistance due to the stray magnetic field from magnetic nanoparticles, a quantitative method for determining the amount of captured biomarkers is proposed [139].

Scientists have reported a chip composed of arrays of TMR sensors with MgO as the insulating layer to detect magnetic particles at the single-particle level [140]. This sensor has an elliptical shape with axis lengths of 400 and 100 nm. The authors claim a linear sensor response in a wide magnetic field range (i.e., −500 to 500 Oe). They have supported their experimental data with simulation results.

**Figure 17:**
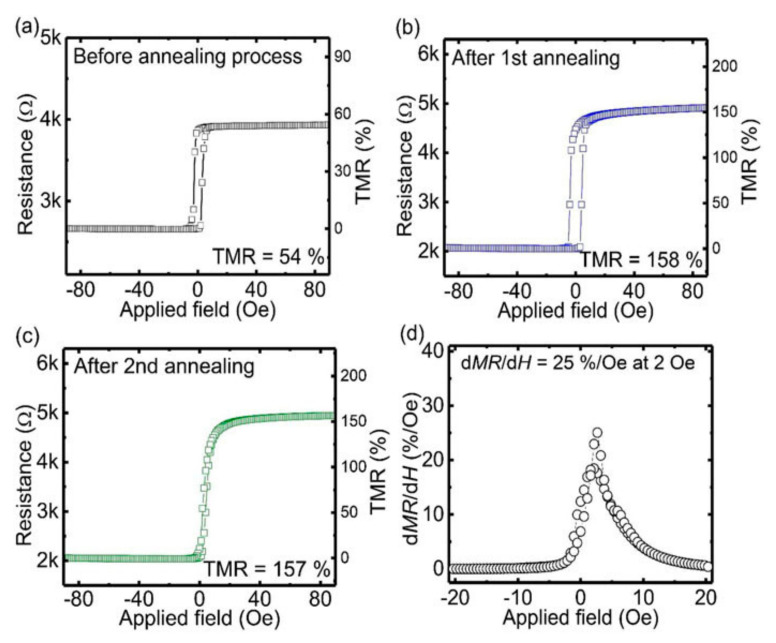
The transfer curves of the TMR sensor proposed in [141] (**a**) before the annealing process, (**b**) after the first annealing, with an applied magnetic field of 10 kOe at 350°C, (**c**) after the second annealing process, with an applied magnetic field of 10 kOe at 300 °C, and (**d**) the dMR/dH dependence on the applied magnetic field are plotted. Reprinted with permission from Z. Jin et al., 2021 AIP Advances 11(1) [141], under a Creative Commons Attribution (CC BY) license. Copyright 2021 AIP Publishing.

A TMR sensor for detecting magnetic nanoparticles operating at low magnetic fields is recently introduced [141]. This sensor with a synthetic antiferromagnetic free layer shows sensitivities of over 18%/Oe at a magnetic field range of 0 to 3 Oe. The authors report improvement of TMR ratio and linearity by twice annealing the sensors using orthogonal magnetic field directions at different temperatures. Figure 17 shows the transfer curves of the proposed sensor in the as-deposited state, after the first annealing and after the second annealing, where the TMR ratios of 52%, 150%, and 160% are achieved, respectively.

Researchers have recently developed a rapid bacteria detection method based on a TMR sensor [142]. To measure Escherichia coli bacteria, they labeled the target by magnetic particles producing a magnetic fringe field in an external magnetic field, the signal of which is detected by the TMR sensor. 

Another group has used TMR sensors and magnetic nanoparticles to detect ricin [143]. They have combined the magnetic immuno-chromatographic test strip and the TMR sensor and claim that it overcomes the challenges that arise in traditional biosensors based on optical signal detection. They also claim it has advantages of easy operation, high sensitivity, reproducibility, and specificity. 

Scientists have used a combination of highly sensitive TMR sensors, magnetic nanoparticles, and microfluidic channels for detecting pathogens in food [144]. They have used the sensor for detecting hybridization of genomic DNA extracted from the pathogenic bacterium Listeria monocytogenes with a sensitivity below the nM range. They first hybridized the chips with complementary target DNA and put it in the microfluidic chip. Then, they injected the streptavidin-coated magnetic nanoparticles with a diameter of 250 nm into the chip. Next, they gave the nanoparticles time to interact with the biotinylated DNA on the sensor surface and recorded the sensor output signal. During the experiment, an external magnetic field magnetized the magnetic nanoparticles.

One of the amazing works in this field is the one by Ikeda and coworkers [145]. They have reported a TMR ratio of 604% at 300 K in Ta∕Co20Fe60B20∕MgO∕Co20Fe60B20∕Ta magnetic tunnel junction. The key point in obtaining a high TMR ratio is the structure annealing at high temperatures (i.e., above 500 °C). They also reported the highest TMR ratio of 1144% at 5 K.

### 5.4. Magnetorelaxometry-Based Sensors

**Figure 18:**
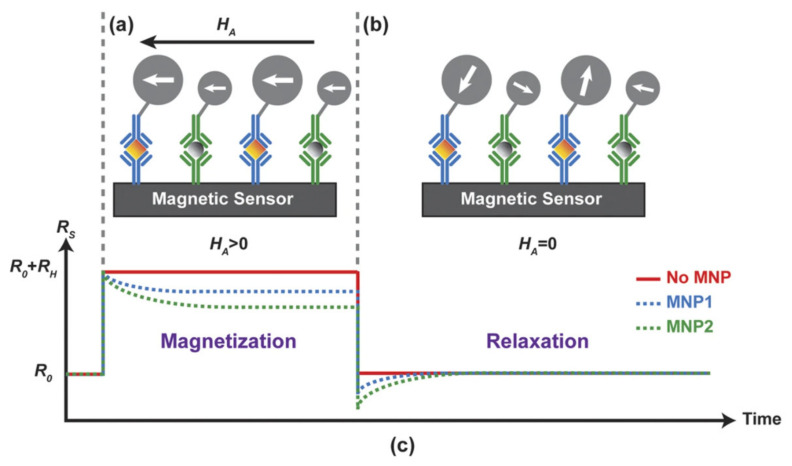
A schematic of an MRX sensor is shown. (**a**) In the magnetization phase, the magnetic moment of the magnetic nanoparticles is aligned to the external field. (**b**) In the relaxation phase, the mag-netic moments of the magnetic nanoparticles are randomized. (**c**) The resistance of the sensor in the magnetization and relaxation phases with and without nanoparticles are illustrated. Re-printed with permission from C. Huang et al., 2017. Scientific Reports 7, 45493 [148]. Copyright 2017 Springer Nature, under a Creative Commons Attribution 4.0 International License http://creativecommons.org/licenses/by/4.0/, accessed on 28 June 2021.

In magnetorelaxometry (MRX), first, magnetic particles are exposed to an external magnetic field. Then, the field is switched off, and the relaxation behavior of the magnetic particles reveals their magnetic properties (see Figure 18). The dynamics of the magnetic nanoparticle magnetic moments are described by Brownian and the Néel relaxations [146,147]. The Brownian relaxation time constant can be written as

(12) $$\tau_{B}=\frac{3\eta V_{h}}{k_{B}T}$$

where *η* and *V_h_* stand for viscosity and the hydrodynamic volume of the particle, respectively. Moreover, the zero-field Néel relaxation time constant is defined by Equation (13):

(13) $$\tau_{N}=\tau_{0}e^{\frac{KV_{c}}{k_{B}T}}$$

where *τ*_0_, *K*, and *V_c_* stand for the damping time, effective magnetic anisotropy, and the volume of the particle core, respectively. If we define the effective relaxation time as $\tau =\frac{\tau_{B}\tau_{N}}{\tau_{B}+\tau_{N}}$, the time-dependent net magnetic flux density can be described as Equation (14)

(14) $$B\left(t\right)=B_{0}e^{-\frac{t}{\tau }}+B_{Offset}$$

where *B*_0_, *t*, and *B_Offset_* stand for the magnetic field amplitude at relaxation time, the time, and the offset, respectively [146].

MRX sensors can measure the magnetic properties of nanoparticles with different methods, including superconducting quantum interference device (SQUID) [146,149], which is typically considered as one of the most sensitive magnetometers [150]. Detection of nanoparticles with SQUID sensors has many applications in biology and immunology [151,152]. Researchers at the University of California at Berkeley claim that in the MRX sensors using SQUID technique, due to the limitations of the magnets and the readout electronics, a several hundred microseconds delay is seen after the applied magnetic field is switched off until the magnetic nanoparticles are detected [153]. To answer this problem, they have reported a Complementary Metal Oxide Semiconductor (CMOS) Hall-effect MRX platform integrating tiny Hall-effect sensors, electromagnets, and high-speed electronic circuits. The authors have tested their system with three particle samples, including magnetic nanoparticles with diameters of 20 and 25 nm with oleic acid coating and magnetic beads with a diameter of 1 µm. They claim this sensor reports various output signals for the three particle sets, minimizes the delay time to less than 100 ns, and its integration with microfluidics makes it a good candidate for lab-on-a-chip applications. However, Hall sensors are used in other magnetometer chips too. In another work, researchers have reported another magnetometer based on the Hall-effect for characterizing magnetic nanoparticles [154]. The proposed chip consists of Hall sensors, transistor switches, and amplifiers. The authors have measured magnetic susceptibility and magnetic moments of magnetic nanoparticles. They claim that measuring the magnetic responses over an external magnetic field range has allowed them to identify magnetic nanoparticle types and their fractional ratios in a mixture. They also have used their system to profile single cancer cells magnetically labeled with magnetic nanoparticles. Moreover, nanoparticles in nano-droplets created with droplet microfluidics are reported with other groups to be detected using Hall sensors [155].

Another group of the proposed MRX sensors is based on GMR [148]. Researchers have measured the signal dependence on the applied magnetic field, the magnetization time, and the particle magnetic core size to study the MRX sensor output. They claim that the achieved characteristic times for various magnetic nanoparticles are different, allowing them to distinguish the particles. 

### 5.5. Other Magnetic Sensors

In addition to the mentioned methods, some other interesting techniques for measuring or detecting magnetic nanoparticles are also available. Researchers have demonstrated accurate quantity characterization of magnetic nanoparticles in a microfluidic chip, based on their effect on the inductance of micro-coils (i.e., magnetic search coil (MSC)) [156]. The magnetic properties of the particles in the microfluidic chamber define the inductance of an electrical resonant circuit in the magnetometer, resulting in a shift in the resonance frequency. More recently, a similar concept is used to detect magnetic materials [157]. The authors show that the rate of the inductance change is proportional to the magnetic permeability and the size of the particles of interest. The authors have investigated this idea both theoretically and experimentally and have achieved errors of less than 9.47%.

Another method for measuring the magnetization of magnetic nanorods is based on the magnetophoresis effect [158]. In this method, the velocity of the nanorods in a microfluidic chip in a magnetic field gradient defines their magnetic properties. The microfluidic channel prevents aggregation of the nanorods and limits their motion in order to analyze them. A similar technique is used for characterizing superparamagnetic beads of different sizes [159]. They claim that their achieved results based on their method agree well with the ones reported by the manufacturer.

Table 4 tabulates the advantages and disadvantages of various sensor types together with some examples.

**Figure 19:**
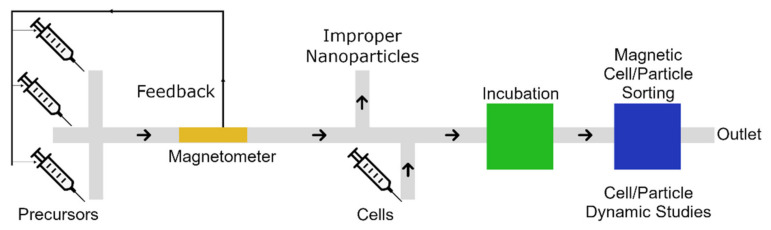
A schematic of a “lab-on-a-chip” device integrating particle synthesis, particle characterization, magnetic labeling, and particle sorting sections on a single chip. In this design, the magnetic nanoparticles are synthesized and enter into the characterization section (yellow area). Based on the signal of the magnetometer a feedback signal is sent to the input controllers to tune the magnetic nanoparticle characteristics. Then, after removing the improper nanoparticles, the synthesized magnetic nanoparticles are used to label the cells in an incubation chamber (green area). Next, the magnetically labeled cells are sorted on-chip (blue area) for biological studies.

Novel “lab-on-a-chip” devices for performing all the required experiments, including nanoparticle synthesis, characterization, magnetic microparticle labeling, and magnetic nano/microparticle sorting and transport, on a single chip, can be developed. Figure 19 illustrates such a microfluidic chip. In this design, we synthesize magnetic nanoparticles using one of the microfluidic-based methods discussed in Section 3. Then, we use one of the magnetometers reviewed in Section 5 to characterize the magnetic nanoparticles and remove the improper ones. Moreover, control feedback signals are sent to the input pumps to modify the particle characteristics and achieve the best possible results. We use proper magnetic nanoparticles to label the living cells in the next step in an incubating chamber. Finally, the magnetic manipulation methods discussed in Section 4 can sort the magnetically labeled cells for further biological studies.

## 6. Conclusions and Future Works (Summary)

In this work, we highlighted the recent fundamental advances in microelectromechanical systems and microfluidic chips used for synthesizing, manipulating, and sensing magnetic nanoparticles. The microfluidic chips can overcome the drawbacks of the conventional nanoparticle synthesis processes. They provide more control over various synthesis parameters with reproducibility, which results in nanoparticles with desired size and morphology. Continuous-flow microreactors are the first microfluidic chips for synthesizing nanoparticles and they are still being used and developed. However, droplet microfluidic chips are the modern tools in this area. They can produce magnetic nanoparticles with narrow size distribution. Microfluidic-based nanoparticle reactors require fewer reagents which makes them cost-efficient competitors for the conventional reactors. 

One major future goal in this field is to synthesize magnetic nanomaterials with microfluidic devices for large-scale applications by potentially using multiple simultaneous microfluidic reactors or other innovative methods. Moreover, there is a great interest in finding techniques for the production of self-assembled magnetic nanoparticle structures. Droplet microfluidic chips have already synthesized the core-shell magnetic nanoparticles; however, the field is still in its infancy, and more advancements for producing magnetic nanoparticles with arbitrary composition, size, and morphologies are needed.

We also discussed various magnetic manipulation techniques. We reviewed some advances in the field, including the works in which permanent magnets, external coils, or embedded micro-coils are used. The magnetic particle techniques based on microfluidics with thin magnetic films are innovative tools for various biomedical applications. Among these methods, magnetophoretic circuits show great advantages such as automation, parallelization, and precise particle transport as single-particle resolution. In addition to manipulating magnetic nanoparticles, the transport of microparticles labeled with magnetic nanoparticles is of great interest.

The field of magnetic particle manipulation is moving fast in the lab-on-a-chip discipline. However, there are some challenges to be answered in the future. In applications where weak magnetic forces are produced, the interaction between the particles and the chip surface becomes problematic. Thus, chip surface passivation and providing a perfect non-fouling layer is considered as one of the main challenges in the field. Moreover, particle manipulation at single-particle resolution is only achieved in the magnetophoretic circuit. Thus, future research needs such magnetic control at a lower cost.

Magnetic sensors to detect magnetic nanoparticles and measure their magnetization were also discussed. The magnetoresistive sensors are relatively low-cost and highly sensitive over a wide frequency range. Thus, they will answer the requirements in fields, such as biology and immunology. The magnetic sensors are widely used in immunoassays, where magnetic nanoparticles bounded to the analyte of interest and immobilized on the sensor surface are detected. The number of detected nanoparticles shows the abundance of the target analyte. We also discussed sensors used for measuring the magnetic properties of the particles. The potential to integrate these sensors with microfluidic-based nanoparticle reactors makes these sensors good candidates for online characterization purposes.

One of the best MR sensors with a high MR value is the TMR sensor class; however, they suffer from large noise. In the future, this challenge needs to be answered. The detectability of the GMR sensors can be enhanced with multilayer structures; however, it increases the fabrication challenges. Moreover, although some sensors with the ability to detect single particles are already proposed, detecting magnetic nanoparticles with low magnetization is still challenging. Thus, more work is needed to increase the sensor sensitivities, with crucial applications in single biomolecule detection.

Overall, the two fields of microelectromechanical systems and nanotechnology are moving at tremendous speeds. They both represent modern technologies with an interesting interface. In the future, we will see a lot of works in which lab-on-a-chip devices are used to further enhance the field of nanotechnology and its applications.

## Figures and Tables

**Table 1:** Examples of microfluidic-based magnetic nanoparticle synthesizes.

Materials	Method	Size (nm)	Ref.
Fe_3_O_4_	Continuous-flow	5–6	[59,60]
Fe_3_O_4_	Droplet-based	4	[57]
Dextran coated Fe_3_O_4_	Continuous-flow	10	[35]
CoFe_2_O_4_	Continuous-flow	6	[61]
Fe_2_O_3_	Continuous-flow	6	[62]
Fe_2_O_3_	Droplet-based	4.7–6	[43]
γ-Fe_2_O_3_@SiO_2_	Continuous-flow	50	[63]
Iron oxide @ chitosan	Continuous-flow	190	[31]
Iron oxide @ chitosan	Droplet-based	104	[56]

**Table 2:** Advantages and challenges of various nanoparticle synthesis methods.

Synthesis Method	Advantages	Challenges
Conventional reactors	- Simple setup	- Low efficiency; - Poor control on parameters; - Low reproducibility; - Agglomeration.
Continuous-flow microreactors	- Simple design; - Relatively simple fabrication; - High throughput; - Good control over parameters, and change of parameters in microseconds; - Sufficient millisecond mixing; - Uniform particle size; - High reproducibility; - Large surface/volume ratio; - Low sample consumption (as low as nanoliters); - Potential for non-spherical particle synthesis; - Potential for automation.	- Channel clogging; - Limitation for heat required protocols; - Taylor dispersion effect; - Poor solvent compatibility; - Sometimes expensive tools.
Droplet-based microreactors	- High throughput (thousands per second); - Uniform and tunable particle size, with polydispersity index as low as 0.024 [73] and sizes of 3.6 nm up to the micrometer range; - Excellent control over parameters, and change of parameters in microseconds; - Sufficient millisecond mixing, followed by as low as 2 ms particle formation; - Very high reproducibility; - Large surface/volume ratio; - Very low sample consumption, (as low as picoliters); - Potential for the synthesis of complicated particles with shells; - Enclosed reaction environment; - Potential for automation.	- Poor solvent compatibility; - Sometimes expensive tools.

**Table 3:** Advantages and challenges of various particle manipulation methods.

Manipulation Method	Advantages	Disadvantages
With external permanent magnet or electromagnet	- Simple design; - Low price, (as low as a few USD).	- Lack of precise control.
With embedded micro-wires and micro-coils	- Control on particles in various sections of the chip; - Independent of external coils or magnets.	- Lack of precise control over individual particles; - Complicated wiring system.
With embedded magnetic thin films	- Precise control over individual particles; - Potential to manipulate many individual particles in parallel (thousands of particles); - Potential for automation; - High throughput.	- Sometimes expensive (a few hundred USD).

**Table 4:** Advantages and disadvantages of multiple magnetic sensors.

Sensor	Advantages	Disadvantages	Examples
AMR	- Small operating field; - Linear operation; - Simple fabrication.	- Fragile at high temperatures; - Low MR ratio.	- 50 nm Fe_3_O_4_ chitosan nanoparticles were detected (detection limit: magnetic moments of 0.56 μemu) [160]. - 50 nm Fe_3_O_4_ chitosan nanoparticles were detected (detection limit of 1 μemu) [127].
GMR	- Moderate MR ratio; - Simple fabrication; - Linear operation.	- Noise at low frequencies.	- 100 nm FeCo nanoparticles were used for detecting DNA with sensitivity of 10 pM [161]. - 200 nm magnetic/polymer beads were used to detect proteins (detection limitation of 7.4 pg/mL) [134]. - 12.8 nm FeCo nanoparticles were used for detecting endoglin (as few as 1000 copies and concentration of 83 fM) [162]. - 4.5 μm beads were detected (as few as 10 beads) [132]. - 50nm Fe_2_O_3_ nanoparticles were to detect Immunoglobulin G protein (140 ng/mL limitation) [163].
TMR	- High MR value; - Low power consumption.	- Large noise; - Complicated fabrication.	- 250 nm streptavidin coated magnetic beads were used to detect DNA (sensitivity below the nM range) [144]. - 16 nm and 50 nm magnetic nanoparticles were used to detect 2.5 μM DNA with signal-to-noise ratios of 25 and 12, respectively [164]. - 200 nm Fe_3_O_4_ beads were detected (sensitivity to detect 500 ng at concentrations of 0.01 mg/m) [141].
MRX	- Avoids the high dynamic range requirement; - Potential for detecting two different particles.		- Fe_3_O_4_ magnetic nanoparticles were detected (volume of 150μL, 100 nmol Fe) [165]. - 22.4 nm Fe_3_O_4_ magnetic nanoparticles were used to detect breast cancer cells (with ability to detect fewer than 100 thousands cells) [166].
